# Baicalein antagonizes rotenone-induced apoptosis in dopaminergic SH-SY5Y cells related to Parkinsonism

**DOI:** 10.1186/1749-8546-7-1

**Published:** 2012-01-21

**Authors:** Ju-Xian Song, Mandy Yuen-Man Choi, Kavin Chun-Kit Wong, Winkie Wing-Yan Chung, Stephen Cho-Wing Sze, Tzi-Bun Ng, Kalin Yan-Bo Zhang

**Affiliations:** 1 School of Chinese Medicine, The University of Hong Kong, 10 Sassoon Road, Pokfulam, Hong Kong SAR, China; 2 School of Biomedical Sciences, The Chinese University of Hong Kong, Shatin, New Territories, Hong Kong, SAR, China

## Abstract

**Background:**

Two active compounds, baicalein and its glycoside baicalin were found in the dried root of *Scutellaria baicalensis *Georgi, and reported to be neuroprotective *in vitro *and *in vivo*. This study aims to evaluate the protective effects of baicalein on the rotenone-induced apoptosis in dopaminergic SH-SY5Y cells related to parkinsonism.

**Methods:**

Cell viability and cytotoxicity were determined by MTT assay. The degree of nuclear apoptosis was evaluated with a fluorescent DNA-binding probe Hoechst 33258. The production of reactive oxidative species (ROS) and loss of mitochondrial membrane potential (ΔΨm) were determined by fluorescent staining with DCFH-DA and Rhodanmine 123, respectively. The expression of Bax, Bcl-2, cleaved caspase-3 and phosphorylated ERK1/2 was determined by the Western blots.

**Results:**

Baicalein significantly increased viability and decreased rotenone-induced death of SH-SY5Y cells in a dose-dependent manner. Pre- and subsequent co-treatment with baicalein preserved the cell morphology and attenuated the nuclear apoptotic characteristics triggered by rotenone. Baicalein antagonized rotenone-induced overproduction of ROS, loss of ΔΨm, the increased expression of Bax, cleaved caspase-3 and phosphorylated ERK1/2 and the decreased expression of Bcl-2.

**Conclusion:**

The antioxidative effect, mitochondrial protection and modulation of anti-and pro-apoptotic proteins are related to the neuroprotective effects of baicalein against rotenone induced cell death in SH-SY5Y cells.

## Background

Parkinson's disease (PD) is a neurodegenerative disease mainly characterized by loss of dopaminergic neurons in the substantia nigra pars compacta [1]. Although the pathology of PD is not understood well, the neurotoxic animal models of PD represent some key neurobehavioral or pathological features [2]. Three neurotoxins, 6-hydroxydopamine (6-OHDA), 1-methyl-4-phenyl-1,2,3,6-tetrahydropyridine (MPTP) and rotenone, are the agents to induce parkinsonism *in vitro *and *in vivo *[3]. An extensive study of these models defined important cellular actions of cell death and offered a basis for the development of novel therapeutic strategies [4]. Rotenone, a lipophilic pesticide, can cross cell membrane easily to induce systemic inhibition of mitochondrial complex I and cause selective nigrostriatal dopaminergic degeneration [5]. Rotenone-induced apoptosis in human neuroblastoma SH-SY5Y cells was mediated by the generation of mitochondrial reactive oxygen species (ROS) [6].

The rotenone model of PD has been used for identifying potential neuroprotective agents in recent years [7]. This model would enable scientific re-evaluation of various herbals for treating PD [8] and facilitate the development of novel anti-parkinsonian drugs [9]. Baicalein and its corresponding glycoside baicalin are two flavonoid compounds found in the dried root of *Scutellaria baicalensis *Georgi. A series of studies demonstrated the neuroprotective effects of baicalein or baicalin in experimental models of Alzheimer's disease [10,11], ischemic stroke [12-15] and PD [16-19]. Baicalein was reported to be effective on 6-OHDA models [18,20,21] and MPTP models [19,22] of PD. This study aims to investigate the neuroprotective effects of baicalein or baicalin on rotenone-induced cellular toxicities (SH-SY5Y cells) *in vitro *and *in vivo*.

## Methods

### Materials

**Figure 1:**
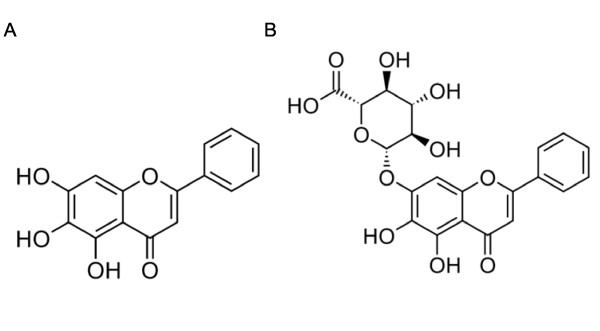
**The chemical structure of (A) baicalein and (B) baicalin**.

Baicalein and baicalin (Figure 1) with purity > 98% were purchased from Shanghai Innovative Research Center of Traditional Chinese Medicine (SIRC/TCM). Stock solutions (100 mM) were prepared in DMSO and diluted with serum-free medium. Dulbecco's Modified Eagle Medium with Nutrient Mixture F-12 (DMEM/F-12), Fetal Bovine Serum (FBS) and Penicillin-Streptomycin were purchased from GIBCO BRL (Grand Island, NY, USA). 2,7-Dichlorofluorescein diacetate (DCFH-DA) and Rhodanmine 123 (Rh123) were purchased from Molecular Probes (Invitrogen, CA, USA). Rotenone, Hoechst 33258, 3-(4,5-dimethylthiazol-2-yl)-2,5-diphenyltetrazolium bromide (MTT), RIPA buffer, BCA Protein Assay Kit and other chemicals were obtained from Sigma-Aldrich (St. Louis, MO, USA). PVDF membrane was purchased from Millipore (MA, USA). Primary antibodies against Bax (D21), Bcl-2 (C21), β-actin and horseradish peroxidase (HRP)-conjugated secondary antibodies were purchased from Santa Cruz Biotechnology (Santa Cruz, CA, USA). Primary antibodies against phospho-p44/42 MAPK (ERK1/2) (Thr202/Tyr204) and cleaved caspase-3 (Asp175) were purchased from Cell Signaling (Beverly, MA, USA). ECL™ Western blotting detection system was purchased from Amersham Biosciences (Piscataway, NJ, USA).

### Cell culture and treatments

Human neuroblastoma SH-SY5Y cells (passage ≤ 25) were cultured as described in our previous study [21] and then treated with different concentrations of rotenone, baicalein or baicalin respectively in serum-free medium for 24 hours to determine their cytotoxicity. To evaluate the protective effects, we pretreated SH-SY5Y cells with different concentrations of baicalein or baicalin for 1 hour and subsequently rotenone was added to the cells for another 24 hours. The final concentration of DMSO in the medium was 0.5%, and showed no cytotoxicity to the cells.

### MTT assay

SH-SY5Y cells seeded on 96-well plates at 80-90% confluence were used in the MTT assay as described in our previous study [21]. In brief, the medium was removed after the treatment. MTT solution (50 μl, 0.5 mg/ml in DMEM/F12) was added to each well and incubated for 4 hours at 37°C. MTT lysis buffer containing 50 μl of 20% SDS (sodium dodecyl sulfate), 50% DMF (N, N-dimethylformamide), adjusted to pH4.7 by HCl (hydrogen chloride) was then added before overnight incubation of the cells at 37°C to dissolve formazan. The absorbance at 570 nm was measured by a microplate reader (Model 680, Bio-Rad Laboratories, UK). The cell viability was expressed as percentage of the control.

### Cellular morphology and nuclear apoptosis

SH-SY5Y cells were incubated with different concentrations of baicalein in serum-free medium for 1 hour, followed by the co-treatment with rotenone (20 μM) for another 24 hours. Chromosomal DNA was stained with a fluorescent DNA-binding probe Hoechst 33258 (5 μg/ml) for 5 minutes, washed with PBS and then observed by a Axiovert S-100 Zeiss fluorescent microscope (Carl Zeiss, Zürich, Switzerland) at 20×. The morphological changes were visualized by phase-contrast imaging at 20×.

### ROS and mitochondrial membrane potential

SH-SY5Y cells were pretreated with different concentrations of baicalein for 1 hour and then co-treated with rotenone (20 μM) for another 6 hours in serum-free medium. According to the protocols described in our previous study [21], the fluorescent probes DCFH-DA and Rh123 were used to determine the generation of intracellular ROS and mitochondrial membrane potential (ΔΨm), respectively. The total cell counts and fluorescent intensity were calculated with Image J software (ImageJ 1.45, http://rsbweb.nih.gov/ij). Mean fluorescent intensity (MFI) was calculated for each group using the following formula:

MFI=total fluorescent intensity×100/total cell counts

### Western blots analysis

SH-SY5Y cells were pre-incubated for 1 hour with different concentrations of baicalein and then co-treated with rotenone (20 μM) for another 24 hours in serum-free medium. Total proteins were extracted using RIPA buffer. Protein determination was by a BCA Protein Assay Kit. Denatured proteins (30 μg) were size fractionated by 12.5% SDS-polyacrylamide gels. Proteins were transferred to PVDF membrane at 80 V for 3 hours. The blots were blocked for 1 hour at room temperature in fresh blocking buffer (0.1% Tween-20 in Tris-buffered saline, pH7.4, containing 5% BSA). The membrane was incubated overnight at 4°C with primary antibodies against Bax, Bcl-2, cleaved caspase-3 and phosphorylated ERK1/2 at dilution of 1:1000. β-actin was used as a loading control. The membrane was incubated for 2 hours with HRP-conjugated secondary antibodies at a dilution of 1:2000. Signals were detected using ECL™ Western blotting detection system. Protein bands were semi-quantified by densitometric analysis using Image J software.

### Statistical analysis

Each experiment was performed at least three times, and the results were presented as means or means ± standard deviations (SD). One-way analysis of variance (ANOVA) followed by Student-Newman-Keuls test for multiple comparison was performed using the SigmaPlot 11.0 software packages (Systat Software Inc., San Jose, CA, USA). Exact *P *values were unavailable due to the software features (Additional file 1 provides a screen snapshot for example). Dose dependence was visually determined from the dose-response graphs. A probability value of *P *< 0.05 was considered to be statistically significant.

## Results

In this study, we evaluated the effects of baicalein and baicalin on rotenone-induced cell death, nuclear apoptosis, production of intracellular ROS, loss of ΔΨm, expressions of Bax, Bcl-2 and caspase-3, and phosphorylation of ERK1/2 in SH-SY5Y cells.

### Cell death

**Figure 2:**
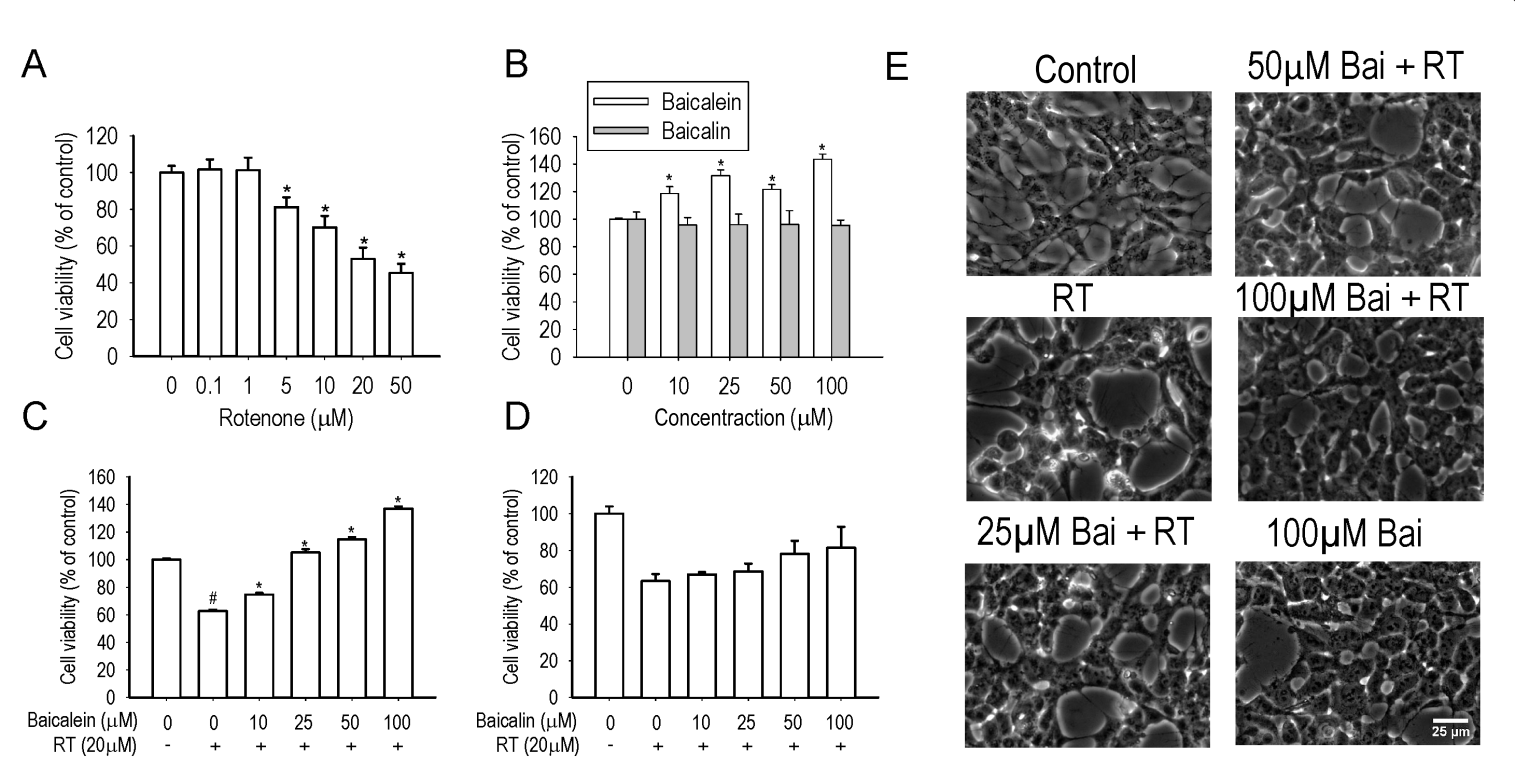
**Effects of baicalein and baicalin on rotenone-induced cell death in SH-SY5Y cells**. Cells were incubated with increasing concentrations of rotenone (A), baicalein and baicalin (B) respectively for 24 hours in serum-free medium (n = 6, *P < 0.01 *versus *control). Cells were pretreated with baicalein (C) or baicalin (D) for 1 hour and then cotreated with 20 μM rotenone for 24 hours in serum-free medium (n = 6, ^#^P < 0.01 *versus *control, *P < 0.01 *versus *rotenone treatment). E: The morphological change was visualized by phase-contrast imaging. Scale bar: 50 μm.

The cytotoxicity of rotenone, baicalein and baicalin were determined by MTT assay, Figure 2A shows that the cell viability was decreased in a dose-dependent manner (*P *< 0.01) by the treatment with rotenone for 24 hours. Rotenone (20 μM) triggered about 50% cell death and this concentration was chosen for subsequent experiments. Both baicalein and baicalin showed no cytotoxicity at the concentrations ranged 10-100 μM. Figure 2B shows that baicalein increased cell viability by 20-40% (*P *< 0.01) at all the tested concentrations, compared with the control.

The effect of baicalein and baicalin on rotenone-induced cell death was evaluated. Figures 2C-D show that pre- and subsequent co-treatment of baicalein significantly inhibited rotenone-induced cell death in a dose-dependent manner (*P *< 0.01). Baicalein (25-100 μM) increased the cell viability up to or even more than the control level (*P *< 0.01). In consistency with the MTT result, the morphological observations revealed that baicalein significantly reversed the cellular damage triggered by rotenone, as shown in Figure 2E. However, baicalin showed no statistically significant protective effect against rotenone-induced cell death.

### Nuclear apoptosis

**Figure 3:**
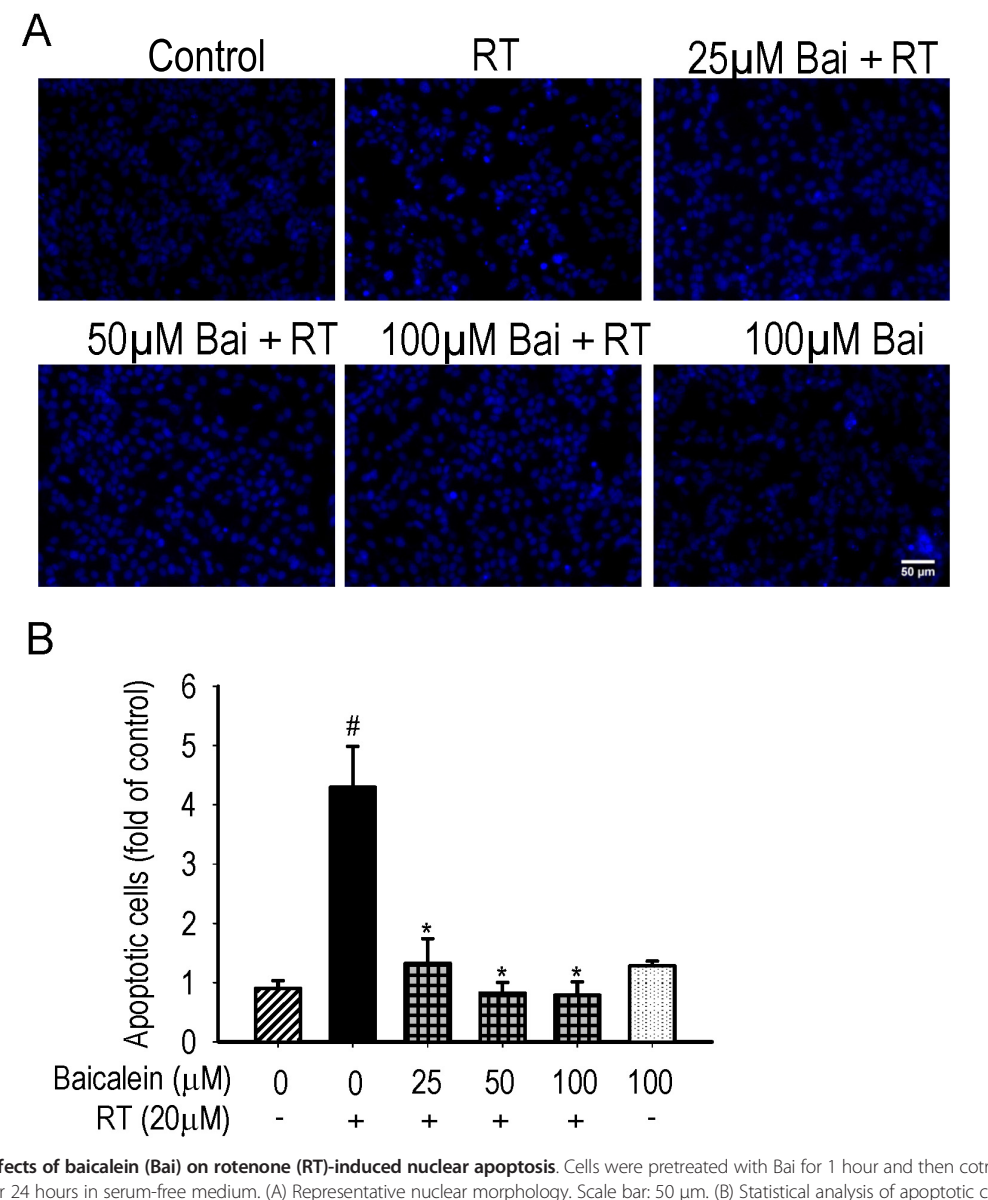
**Effects of baicalein (Bai) on rotenone (RT)-induced nuclear apoptosis**. Cells were pretreated with Bai for 1 hour and then cotreated with 20 μM RT for 24 hours in serum-free medium. (A) Representative nuclear morphology. Scale bar: 50 μm. (B) Statistical analysis of apoptotic cells. At least 600 randomly selected cells were counted in each experiment (n = 3, ^#^P < 0.01 *versus *control, *P < 0.01 *versus *RT treatment).

Compared with the control, the apoptotic characteristics induced by the rotenone treatment, such as nuclear condensation and fragmentation, could be attenuated by pre- and subsequent co-treatment with increasing concentrations of baicalein (as shown in Figure 3). The statistical data showed 4.29 ± 0.69 folds of increase in the ratio of apoptotic cells triggered by rotenone, which could be reduced to the control level by pre- and subsequent co-treatment with increasing concentrations of baicalein (*P *< 0.01). Baicalein treatment for 24 hours had no significantly effect on nuclear apoptosis.

### Intracellular ROS

**Figure 4:**
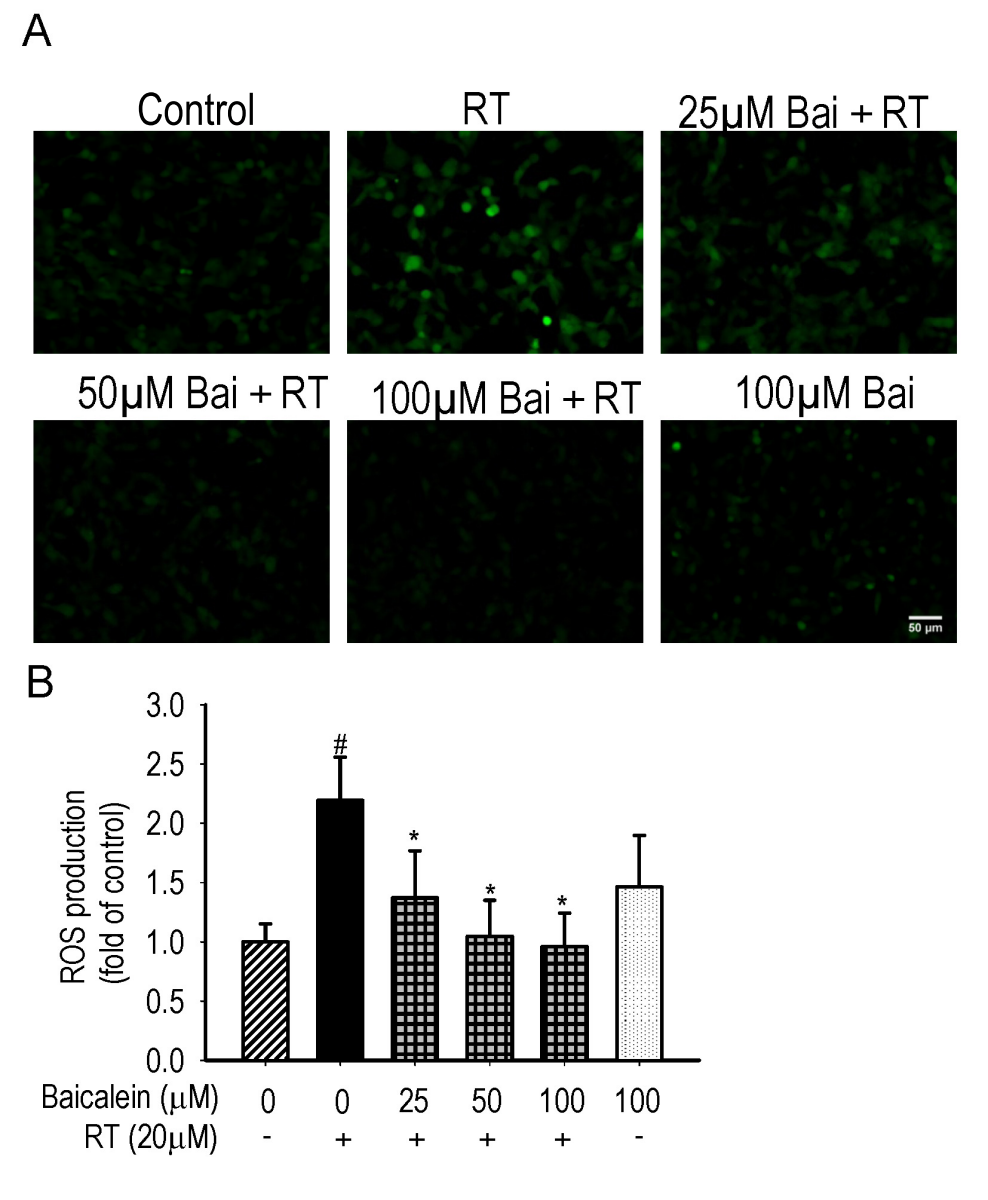
**Effects of baicalein (Bai) on rotenone (RT)-induced ROS overproduction**. Cells were pretreated with Bai for 1 hour and then cotreated with 20 μM RT for 6 hours in serum-free medium. The ROS generation was determined by the mean fluorescent intensity (MFI) of DCFH-DA. (A) Representative fluorescent images. Scale bar: 50 μm. (B) Statistical analysis. At least 600 randomly selected cells were counted in each experiment (n = 3, ^#^P < 0.01 *versus *control, *P < 0.01 *versus *RT treatment).

Figure 4 demonstrates that rotenone treatment induced 2.19 ± 0.36 folds of increase in the intracellular ROS compared with the control (*P *< 0.01). Pre- and subsequent co-treatment with baicalein reduced the production of ROS in a dose-dependent manner (*P *< 0.01) down to the control level. Baicalein treatment for 6 hours showed no significant effect on ROS production as compared with the control.

### Loss of ΔΨm

**Figure 5:**
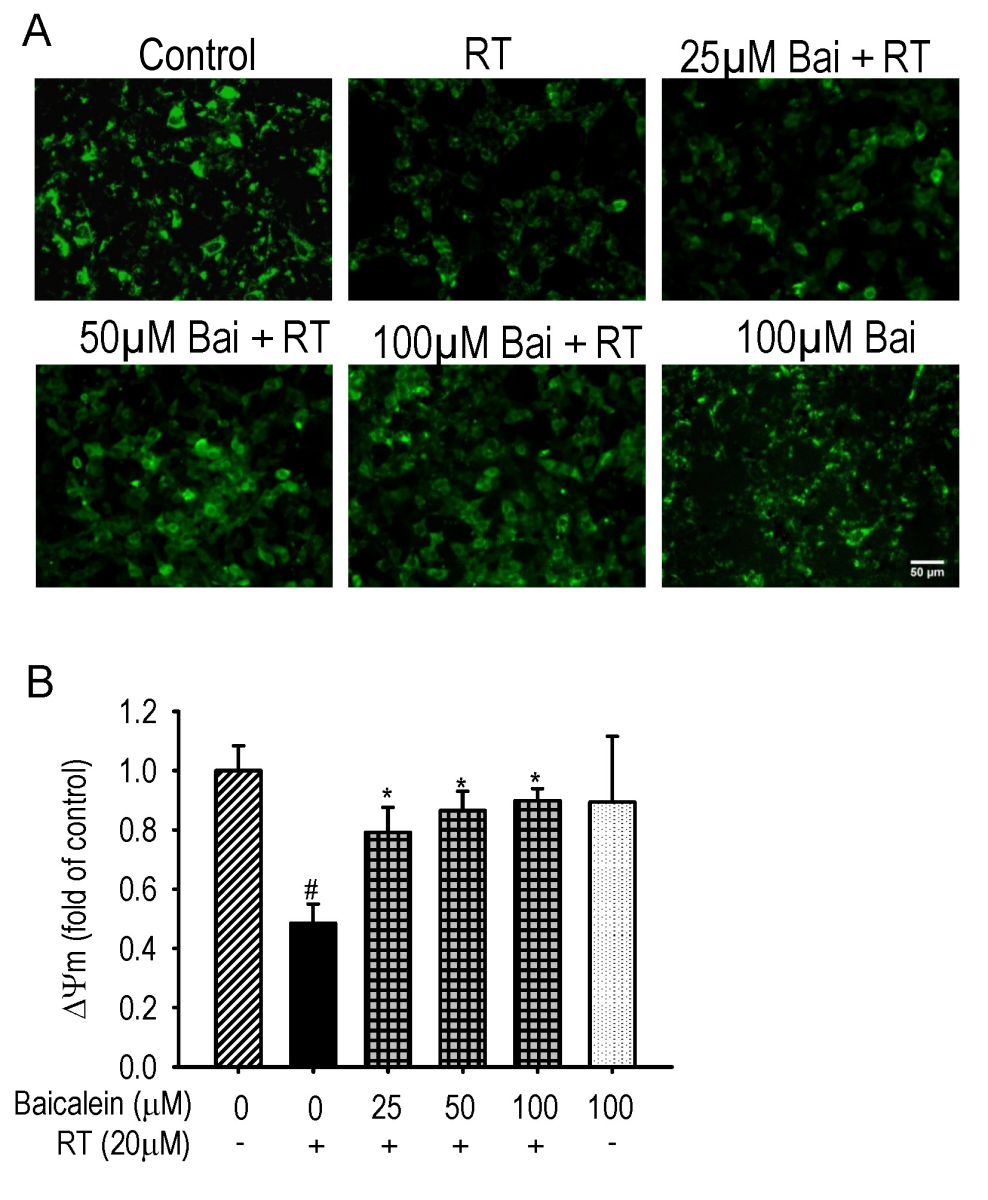
**Effects of baicalein (Bai) on rotenone (RT)-induced loss of ΔΨm**. Cells were pretreated with Bai for 1 hour and then cotreated with 20 μM RT for 6 hours in serum-free medium. The ΔΨm was determined by the mean fluorescent intensity (MFI) of Rh123. (A) Representative fluorescent images. Scale bar: 50 μm. (B) Statistical analysis. At least 600 randomly selected cells were counted in each experiment (n = 3, ^#^P < 0.01 *versus *control, *P < 0.01 *versus *RT treatment).

The inhibition of complex I by rotenone may induce loss of ΔΨm and the release of pro-apoptotic proteins [23]. As shown in Figure 5, rotenone treatment led to about 2 folds of decrease in Rh123 fluorescence (*P *< 0.01), reflecting the loss of ΔΨm. Pre- and subsequent co-treatment with baicalein significantly inhibited the loss of ΔΨm in a dose-dependent manner (*P *< 0.01). Baicalein treatment for 6 hours showed no significant effect on ΔΨm as compared with the control.

### Expression of Bax, Bcl-2 and cleaved caspase-3

**Figure 6:**
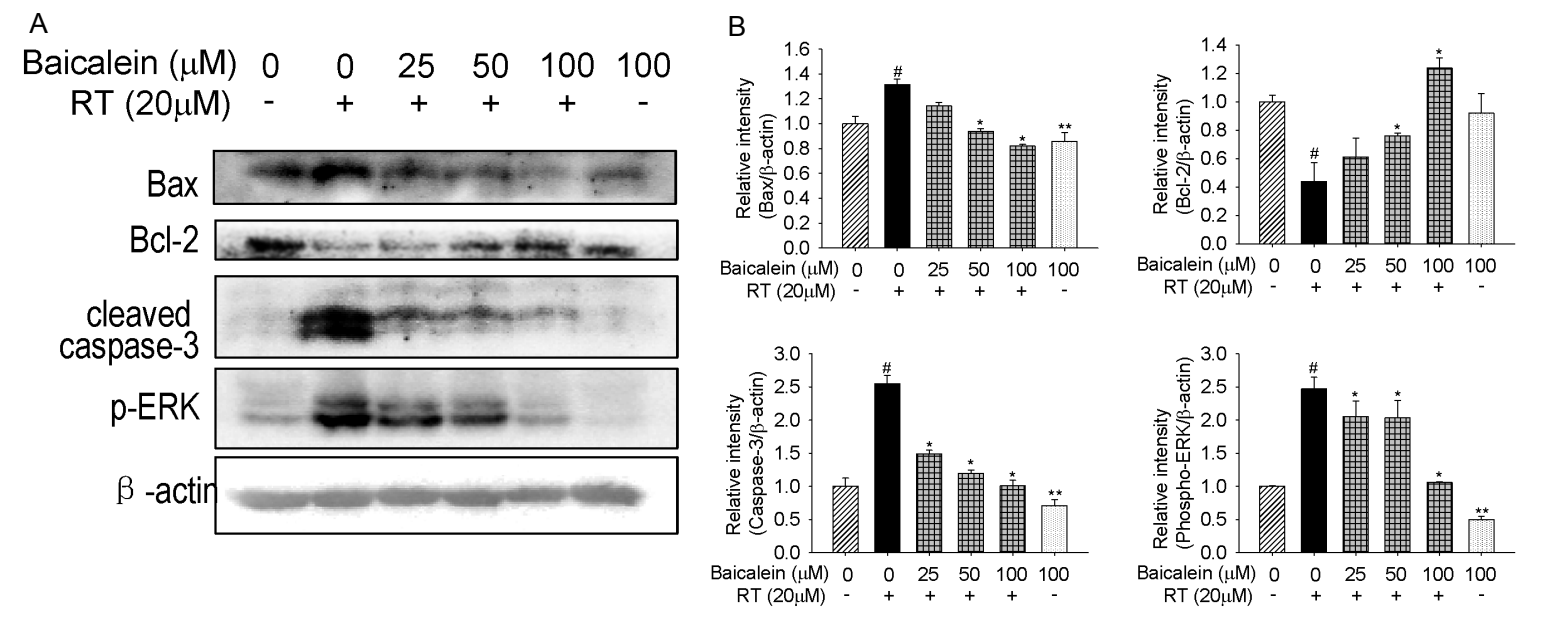
**Effects of baicalein (Bai) on rotenone (RT)-induced imbalance in the expression of Bax, Bcl-2, cleaved caspase-3 and phopho-ERK1/2**. Cells were pretreated with Bai for 1 hour and then cotreated with 20 μM RT for 24 hours in serum-free medium. Blots were stripped and reprobed for β-actin as a loading control. (A) Representative protein bands. (B) Statistical analysis. The corresponding bar graph represented data quantified from three independent experiments (n = 3, ^#^P < 0.05 *versus *control, *P < 0.05 *versus *RT treatment, **P < 0.05 *versus *control).

To further characterize the mechanism of baicalein inhibition on rotenone-induced apoptosis, we determined the effect of baicalein on the expression of anti- and pro-apoptotic proteins by Western blots. As shown in Figure 6, the expression of Bax and cleaved caspase-3 was increased while the expression of Bcl-2 was significantly decreased by the treatment with rotenone (20 μM) for 24 hours (*P *< 0.05), compared with the control. Pre- and subsequent co-treatment with increasing concentrations of baicalein gradually restored the imbalanced expression profile of these proteins. Interestingly, baicalein treatment alone for 24 hours could reduce the base levels of Bax (0.86 ± 0.07) and cleaved caspase-3 (0.71 ± 0.09) (*P *< 0.05).

### ERK1/2 phosphorylation

It was reported that rotenone induced ERK1/2 phosphorylation and neuronal degeneration in hippocampus neurons [24]. Similar to this finding, we detected 2.47 ± 0.18 folds of increase in the expression of phosphorylated ERK1/2 in SH-SY5Y cells by treatment with rotenone for 24 hours, as shown in Figure 6 (*P *< 0.05). Pre- and subsequent co-treatment with baicalein reduced the expression of phosphorylated ERK1/2 down to the control level in a dose-dependent manner. Baicalein treatment alone for 24 hours could also significantly reduce the base level of ERK1/2 phosphorylation.

## Discussion

In the study, we evaluated the neuroprotective effects of baicalein on rotenone-induced SH-SY5Y cell apoptosis. In the neurotoxic models (6-OHDA and MPTP/MPP^+^) of PD, either baicalein or baicalin has been reported to be effective [18,22,25]. However, we found that only baicalein showed a significant inhibition on rotenone-induced cytotoxicity as demonstrated in Figure 2D. Choi *et al*. [26] demonstrated that baicalein was protective against endoplasmic reticulum (ER) stress-induced ROS accumulation and apoptosis. The difference between baicalein and baicalin in antioxidative potential and cellular permeability might contribute to their difference in cytoprotective effects against ER stress-inducers [26]. These two factors may also account for the different effects of baicalein and baicalin on rotenone-induced cytotoxicity.

MTT cell viability assay showed that baicalein antagonized rotenone-induced cell death, which may be due to the ability of baicalein in increasing the cell viability of normal cells, as indicated in Figure 2B. The cell viability was reduced to 62.64% (*P *< 0.01) by treatment with rotenone alone for 24 hours while pre- and subsequent co-treatment with baicalein (100 μM) increased the cell viability to 137.01% (*P *< 0.01), as shown in Figure 2C. Baicalein (100 μM) treatment alone induced 43.46% increase (*P *< 0.01) in cell viability (Figure 2B) and the difference in cell viability (Figure 2C) between rotenone treatment alone (62.64%) and baicalein (100 μM) pre and co-treatment (137.01%) is 74.37%, suggesting that the cell proliferating activity of baicalein (43.46% increase) does not account for its protection against rotenone-induced cell death (74.37% increase). In other words, the protection of baicalein against rotenone-induced cell death may be independent of its cell proliferation activity. These results suggested that baicalein had protection against rotenone-induced cytotoxicity independent of its cell proliferation activity.

Oxidative injury was proposed to be a primary mechanism of mitochondrial toxicity in the rotenone-induced degeneration of dopaminergic neurons [27,28]. Impairment of complex I activity by rotenone led to excess ROS formation, which induced loss of ΔΨm and initiated apoptotic cell death [27,28]. It was reported that baicalein suppressed the mitochondrial dysfunction induced by hydrogen peroxide and 6-OHDA, and the initiation of the loss of ΔΨm in PC12 cells and SH-SY5Y cells, respectively [17,29]. This study confirmed these findings that baicalein inhibited ROS production and loss of ΔΨm triggered by rotenone in SH-SY5Y cells, resulting in cellular resistance against the initiating steps of apoptosis. This protection was mediated in part by its antioxidative ability and preservation of mitochondrial function.

The balance of Bax and Bcl-2 proteins relates to the cell viability [30]. Loss of ΔΨm increases the mitochondrial permeability and results in the release of cytochrome c from the mitochondria, which triggers activation of caspase-9/3 and ultimate cell death [31]. In this study, we found that baicalein restored the imbalance of the expression profiles of Bax, Bcl-2 and cleaved caspase-3; baicalein treatment alone could also decrease the expression of Bax and cleaved caspase-3; and modulation of the pro- and anti-apoptotic proteins would be involved in the protective effects of baicalein against rotenone-induced neurotoxicity.

Sustained ERK activation was reported to promote cell death in neuronal cells treated with neurotoxins [32-34]. Figure 6 demonstrates that rotenone triggering significant phosphorylation and activation of ERK1/2 was antagonized by baicalein pretreatment, indicating that inactivation of ERK1/2 pathway was involved in the neuroprotective effects of baicalein against rotenone-induced neurotoxicity.

## Conclusion

Inhibition of ROS overproduction, preservation of mitochondrial function, modulation of anti- and pro-apoptotic proteins and inactivation of ERK1/2 pathway are related to the neuroprotective effects of baicalein against rotenone-induced apoptosis in dopaminergic SH-SY5Y cells.

## Abbreviations

DCFH-DA: 2,7-Dichlorofluorescein diacetate; DMEM/F-12, Dulbecco's Modified Eagle Medium: Nutrient Mixture F-12; DMF: N, N-dimethylformamide; DMSO: dimethyl sulfoxide; ERK1/2: extracellular signal-regulated kinases 1 and 2; FBS: fetal bovine serum; HCl: hydrogen chloride; HRP: horseradish peroxidase; MAPK: mitogen activated protein kinases; MPP^+^: 1-methyl-4-phenyl pyridinium; MPTP: 1-methyl-4-phenyl-1,2,3,6-tetrahydropyridine; MTT: 3-(4,5-dimethylthiazol-2-yl)- 2,5-diphenyltetrazolium bromide; PD: Parkinson's disease; Rh123: Rhodanmine 123; ROS: reactive oxygen species; SDS: sodium dodecyl sulfate; 6-OHDA: 6-hydroxydopamine; ΔΨm: mitochondrial membrane potential.

## Competing interests

The authors declare that they have no competing interests.

## Authors' contributions

JXS and KYBZ designed the study and drafted the manuscript. JXS, MYMC, KCKW and WWYC conducted the experiments and analyzed the data. SCWS and TBN revised the manuscript. All authors read and approved the final version of the manuscript.

## Supplementary Material

Additional file 1 **A screen snapshot demonstrating the statistical analysis using SigmaPlot 11.0**. The detailed procedures are illustrated for Figure 2C. Exact *P *values were unavailable due to the software features. Click here for file

## References

[B1] Schapira AH Bezard E Brotchie J Calon F Collingridge GL Ferger B Hengerer B Hirsch E Jenner P Le Novere N Obeso JA Schwarzschild MA Spampinato U Davidai G Novel pharmacological targets for the treatment of Parkinson's disease Nat Rev Drug Discov 2006 5 845 854 10.1038/nrd2087 17016425

[B2] Cannon JR Greenamyre JT Neurotoxic *in vivo *models of Parkinson's disease recent advances Prog Brain Res 2010 184 17 33 20887868 10.1016/S0079-6123(10)84002-6

[B3] Beal MF Experimental models of Parkinson's disease Nat Rev Neurosci 2001 2 325 334 11331916 10.1038/35072550

[B4] Fox SH Brotchie JM The MPTP-lesioned non-human primate models of Parkinson's disease. Past, present, and future Prog Brain Res 2010 184 133 157 20887873 10.1016/S0079-6123(10)84007-5

[B5] Betarbet R Sherer TB MacKenzie G Garcia-Osuna M Panov AV Greenamyre JT Chronic systemic pesticide exposure reproduces features of Parkinson's disease Nat Neurosci 2000 3 1301 1306 10.1038/81834 11100151

[B6] Watabe M Nakaki T Mitochondrial complex I inhibitor rotenone-elicited dopamine redistribution from vesicles to cytosol in human dopaminergic SH-SY5Y cells J Pharmacol Exp Ther 2007 323 499 507 10.1124/jpet.107.127597 17726156

[B7] Greenamyre JT Cannon JR Drolet R Mastroberardino PG Lessons from the rotenone model of Parkinson's disease Trends Pharmacol Sci 2010 31 141 142 10.1016/j.tips.2009.12.006 20096940 PMC2846992

[B8] Manyam BV Sanchez-Ramos JR Traditional and complementary therapies in Parkinson's disease Adv Neurol 1999 80 565 574 10410773

[B9] Li Q Zhao D Bezard E Traditional Chinese medicine for Parkinson's disease: a review of Chinese literature Behav Pharmacol 2006 17 403 410 10.1097/00008877-200609000-00006 16940761

[B10] Lebeau A Esclaire F Rostene W Pelaprat D Baicalein protects cortical neurons from beta-amyloid (25-35) induced toxicity Neuroreport 2001 12 2199 2202 10.1097/00001756-200107200-00031 11447334

[B11] Wang SY Wang HH Chi CW Chen CF Liao JF Effects of baicalein on beta-amyloid peptide-(25-35)-induced amnesia in mice Eur J Pharmacol 2004 506 55 61 10.1016/j.ejphar.2004.10.029 15588624

[B12] Cui L Zhang X Yang R Liu L Wang L Li M Du W Baicalein is neuroprotective in rat MCAO model: role of 12/15-lipoxygenase, mitogen-activated protein kinase and cytosolic phospholipase A2 Pharmacol Biochem Behav 2010 96 469 475 10.1016/j.pbb.2010.07.007 20637223

[B13] Liu C Wu J Xu K Cai F Gu J Ma L Chen J Neuroprotection by baicalein in ischemic brain injury involves PTEN/AKT pathway J Neurochem 2010 112 1500 1512 10.1111/j.1471-4159.2009.06561.x 20050973

[B14] Xue X Qu XJ Yang Y Sheng XH Cheng F Jiang EN Wang JH Bu W Liu ZP Baicalin attenuates focal cerebral ischemic reperfusion injury through inhibition of nuclear factor kappaB p65 activation Biochem Biophys Res Commun 2010 403 398 404 10.1016/j.bbrc.2010.11.042 21093411

[B15] Zhang Z Wu R Li P Liu F Zhang W Zhang P Wang Y Baicalin administration is effective in positive regulation of twenty-four ischemia/reperfusion-related proteins identified by a proteomic study Neurochem Int 2009 54 488 496 10.1016/j.neuint.2009.02.005 19428793

[B16] Jiang M Porat-Shliom Y Pei Z Cheng Y Xiang L Sommers K Li Q Gillardon F Hengerer B Berlinicke C Smith WW Zack DJ Poirier MA Ross CA Duan W Baicalein reduces E46K alpha-synuclein aggregation in vitro and protects cells against E46K alpha-synuclein toxicity in cell models of familiar Parkinsonism J Neurochem 2010 114 419 429 10.1111/j.1471-4159.2010.06752.x 20412383 PMC2910156

[B17] Lee HJ Noh YH Lee DY Kim YS Kim KY Chung YH Lee WB Kim SS Baicalein attenuates 6-hydroxydopamine-induced neurotoxicity in SH-SY5Y cells Eur J Cell Biol 2005 84 897 905 10.1016/j.ejcb.2005.07.003 16323286

[B18] Mu X He G Cheng Y Li X Xu B Du G Baicalein exerts neuroprotective effects in 6-hydroxydopamine-induced experimental parkinsonism in vivo and in vitro Pharmacol Biochem Behav 2009 92 642 648 10.1016/j.pbb.2009.03.008 19327378

[B19] Mu X He GR Yuan X Li XX Du GH Baicalein protects the brain against neuron impairments induced by MPTP in C57BL/6 mice Pharmacol Biochem Behav 2011 98 286 291 10.1016/j.pbb.2011.01.011 21262257

[B20] Im HI Joo WS Nam E Lee ES Hwang YJ Kim YS Baicalein prevents 6-hydroxydopamine-induced dopaminergic dysfunction and lipid peroxidation in mice J Pharmacol Sci 2005 98 185 189 10.1254/jphs.SC0050014 15942123

[B21] Song JX Shaw PC Sze CW Tong Y Yao XS Ng TB Zhang YB Chrysotoxine, a novel bibenzyl compound, inhibits 6-hydroxydopamine induced apoptosis in SH-SY5Y cells via mitochondria protection and NF-kappaB modulation Neurochem Int 2010 57 676 689 10.1016/j.neuint.2010.08.007 20708055

[B22] Cheng Y He G Mu X Zhang T Li X Hu J Xu B Du G Neuroprotective effect of baicalein against MPTP neurotoxicity: behavioral, biochemical and immunohistochemical profile Neurosci Lett 2008 441 16 20 10.1016/j.neulet.2008.05.116 18586394

[B23] Hu LF Lu M Wu ZY Wong PT Bian JS Hydrogen sulfide inhibits rotenone-induced apoptosis via preservation of mitochondrial function Mol Pharmacol 2009 75 27 34 10.1124/mol.108.047985 18832435

[B24] Sai Y Chen J Wu Q Liu H Zhao J Dong Z Phosphorylated-ERK 1/2 and neuronal degeneration induced by rotenone in the hippocampus neurons Environ Toxicol Pharmacol 2009 27 366 372 10.1016/j.etap.2008.12.004 21783966

[B25] Chen X Zhang N Zou HY Protective effect of baicalin on mouse with Parkinson's disease induced by MPTP Zhongguo Zhong Xi Yi Jie He Za Zhi 2007 27 1010 1012 18173149

[B26] Choi JH Choi AY Yoon H Choe W Yoon KS Ha J Yeo EJ Kang I Baicalein protects HT22 murine hippocampal neuronal cells against endoplasmic reticulum stress-induced apoptosis through inhibition of reactive oxygen species production and CHOP induction Exp Mol Med 2010 42 811 822 10.3858/emm.2010.42.12.084 20959717 PMC3015155

[B27] Radad K Rausch WD Gille G Rotenone induces cell death in primary dopaminergic culture by increasing ROS production and inhibiting mitochondrial respiration Neurochem Int 2006 49 379 386 10.1016/j.neuint.2006.02.003 16580092

[B28] Testa CM Sherer TB Greenamyre JT Rotenone induces oxidative stress and dopaminergic neuron damage in organotypic substantia nigra cultures Brain Res Mol Brain Res 2005 134 109 118 15790535 10.1016/j.molbrainres.2004.11.007

[B29] Zhang S Ye J Dong G Neuroprotective effect of baicalein on hydrogen peroxide-mediated oxidative stress and mitochondrial dysfunction in PC12 cells J Mol Neurosci 2009 40 311 320 10.1007/s12031-009-9285-5 19731100

[B30] Borner C The Bcl-2 protein family: sensors and checkpoints for life-or-death decisions Mol Immunol 2003 39 615 647 10.1016/S0161-5890(02)00252-3 12493639

[B31] Chinnaiyan AM Orth K O'Rourke K Duan H Poirier GG Dixit VM Molecular ordering of the cell death pathway. Bcl-2 and Bcl-xL function upstream of the CED-3-like apoptotic proteases J Biol Chem 1996 271 4573 4576 10.1074/jbc.271.9.4573 8617712

[B32] Gomez-Santos C Ferrer I Reiriz J Vinals F Barrachina M Ambrosio S MPP^+ ^increases alpha-synuclein expression and ERK/MAP-kinase phosphorylation in human neuroblastoma SH-SY5Y cells Brain Res 2002 935 32 39 10.1016/S0006-8993(02)02422-8 12062470

[B33] Kulich SM Horbinski C Patel M Chu CT 6-Hydroxydopamine induces mitochondrial ERK activation Free Radic Biol Med 2007 43 372 383 10.1016/j.freeradbiomed.2007.04.028 17602953 PMC2023873

[B34] Zhu JH Horbinski C Guo F Watkins S Uchiyama Y Chu CT Regulation of autophagy by extracellular signal-regulated protein kinases during 1-methyl-4-phenylpyridinium-induced cell death Am J Pathol 2007 170 75 86 10.2353/ajpath.2007.060524 17200184 PMC1762689

