# Supplementary material for: Baicalein antagonizes rotenone-induced apoptosis in dopaminergic SH-SY5Y cells related to Parkinsonism
Source: Chin Med. 2012 Jan 21;7:1. doi: 10.1186/1749-8546-7-1 (PMC3275529; doi:10.1186/1749-8546-7-1)
Supplement: Additional file 1 — A screen snapshot demonstrating the statistical analysis using SigmaPlot 11.0. The detailed procedures are illustrated for Figure 2C. Exact P values were unavailable due to the software features. [file 1749-8546-7-1-S1.PDF]

Example: Fig. 2C

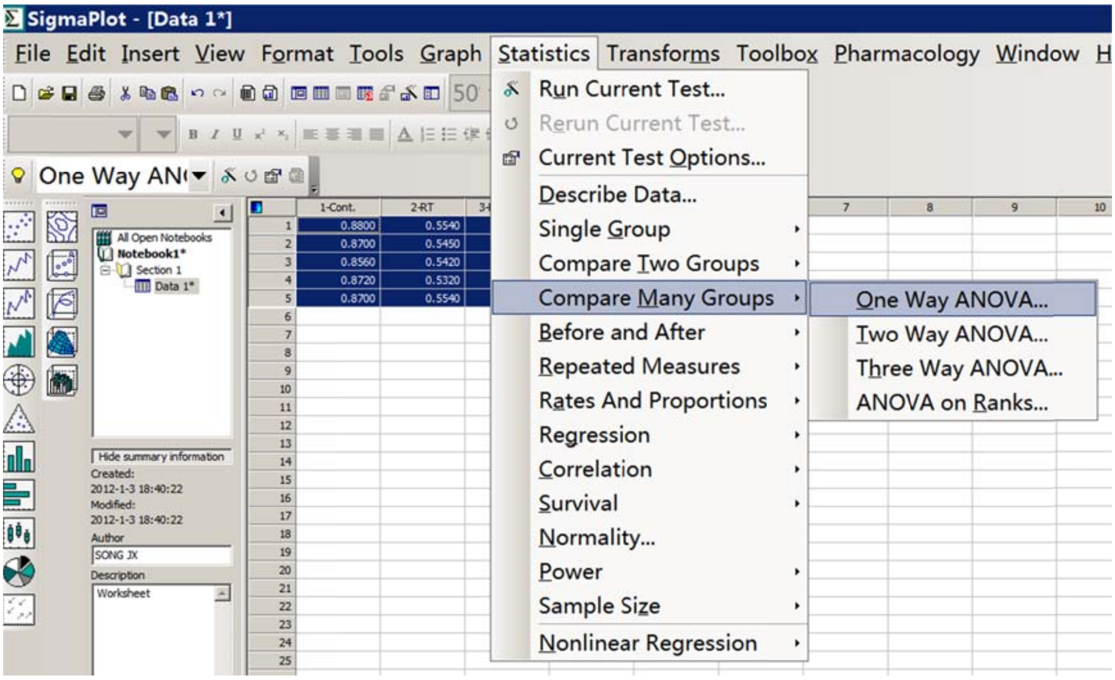

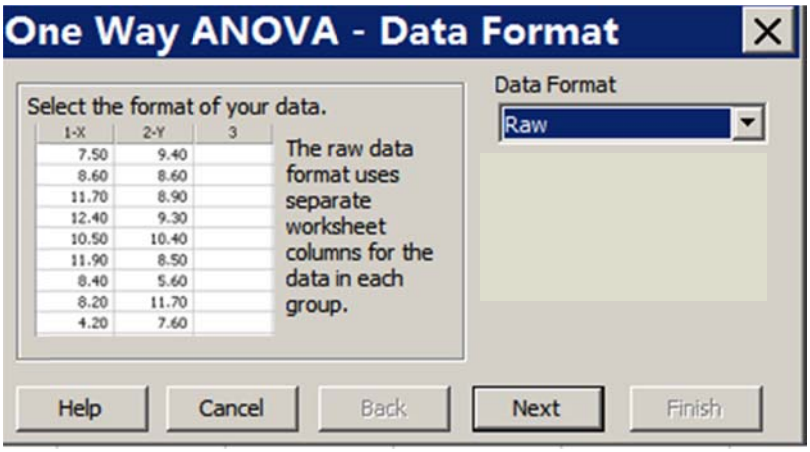

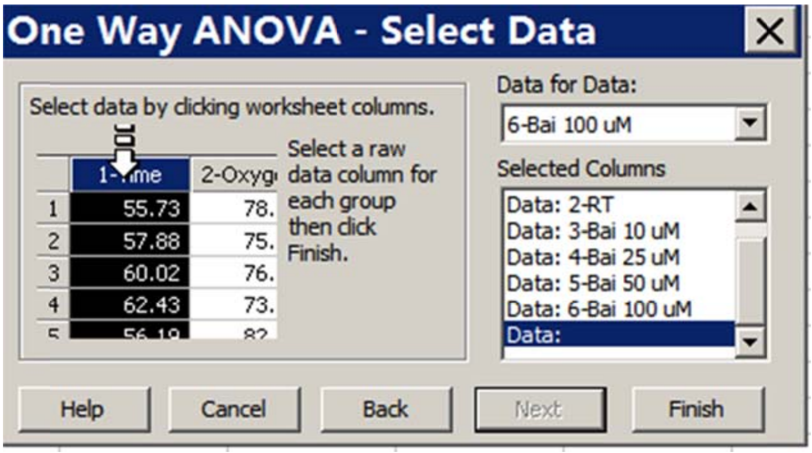

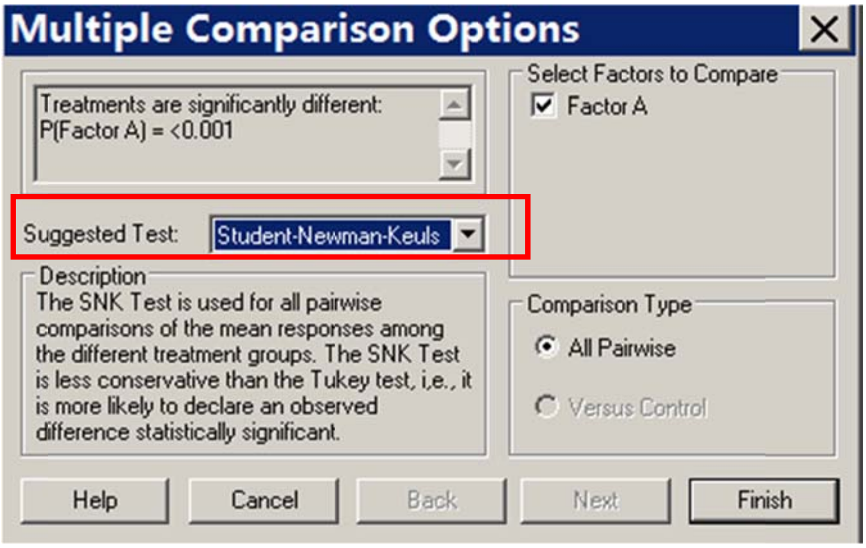

Data source: Data 1 in Notebook1

Normality Test (Shapiro-Wilk) Passed (P = 0.312)

Equal Variance Test: Passed (P = 0.674)

Group Name	N	Missing	Mean	Std Dev	SEM
Cont.	5	0	0.870	0.00865	0.00387
RT	5	0	0.545	0.00921	0.00412
Bai 10 uM	5	0	0.649	0.0110	0.00493
Bai 25 uM	5	0	0.915	0.0215	0.00960
Bai 50 uM	5	0	0.998	0.0137	0.00613
Bai 100 uM	5	0	1.192	0.0135	0.00605

Source of Variation	DF	SS	MS	F	P
Between Groups	5	1.379	0.276	1487.610	<0.001
Residual	24	0.00445	0.000185		
Total	29	1.384			

The differences in the mean values among the treatment groups are greater than would be expected by chance; there is a statistically significant difference (P = <0.001).

Power of performed test with alpha = 0.050: 1.000

All Pairwise Multiple Comparison Procedures (Student-Newman-Keuls Method) :

Comparisons for factor:

Comparison	Diff of Means	p	q	P	P<0.050
Bai 100 uM vs. RT	0.646	6	106.148	<0.001	Yes
Bai 100 uM vs. Bai 10 uM	0.543	5	89.201	<0.001	Yes
Bai 100 uM vs. Cont.	0.322	4	52.910	<0.001	Yes
Bai 100 uM vs. Bai 25 uM	0.277	3	45.487	<0.001	Yes
Bai 100 uM vs. Bai 50 uM	0.194	2	31.858	<0.001	Yes
Bai 50 uM vs. RT	0.452	5	74.290	<0.001	Yes
Bai 50 uM vs. Bai 10 uM	0.349	4	57.344	<0.001	Yes
Bai 50 uM vs. Cont.	0.128	3	21.052	<0.001	Yes
Bai 50 uM vs. Bai 25 uM	0.0830	2	13.630	<0.001	Yes
Bai 25 uM vs. RT	0.369	4	60.661	<0.001	Yes
Bai 25 uM vs. Bai 10 uM	0.266	3	43.714	<0.001	Yes
Bai 25 uM vs. Cont.	0.0452	2	7.422	<0.001	Yes
Cont. vs. RT	0.324	3	53.238	<0.001	Yes
Cont. vs. Bai 10 uM	0.221	2	36.291	<0.001	Yes
Bai 10 uM vs. RT	0.103	2	16.947	<0.001	Yes

the exact P values are not provided
